# Zero-Valent Iron Filtration Reduces Microbial Contaminants in Irrigation Water and Transfer to Raw Agricultural Commodities

**DOI:** 10.3390/microorganisms9102009

**Published:** 2021-09-23

**Authors:** Brienna L. Anderson-Coughlin, Pushpinder K. Litt, Seongyun Kim, Shani Craighead, Alyssa J. Kelly, Pei Chiu, Manan Sharma, Kalmia E. Kniel

**Affiliations:** 1 Department of Animal and Food Science, University of Delaware, Newark, DE 19716, USA; briennaa@udel.edu (B.L.A.-C.); pushpinder87@gmail.com (P.K.L.); scraig@udel.edu (S.C.); alyssake@udel.edu (A.J.K.); 2 Beltsville Agricultural Research Center, Environmental Microbiology and Food Safety Laboratory, United States Department of Agriculture, Agricultural Research Service, Northeast Area, Beltsville, MD 20705, USA; seongyun.kim@usda.gov (S.K.); manan.sharma@ars.usda.gov (M.S.); 3 Department of Civil and Environmental Engineering, University of Delaware, Newark, DE 19716, USA; pei@udel.edu

**Keywords:** irrigation water, *Escherichia coli*, PMMoV, water treatment, zero-valent iron, contamination, microbiological indicators

## Abstract

Groundwater depletion is a critical agricultural irrigation issue, which can be mitigated by supplementation with water of higher microbiological risk, including surface and reclaimed waters, to support irrigation needs in the United States. Zero-valent iron (ZVI) filtration may be an affordable and effective treatment for reducing pathogen contamination during crop irrigation. This study was performed to determine the effects of ZVI filtration on the removal and persistence of *Escherichia coli*, and pepper mild mottle virus (PMMoV) in irrigation water. Water was inoculated with *E. coli* TVS 353, filtered through a ZVI filtration unit, and used to irrigate cucurbit and cruciferous crops. Water (*n* = 168), leaf (*n* = 40), and soil (*n* = 24) samples were collected, the *E. coli* were enumerated, and die-off intervals were calculated for bacteria in irrigation water. Variable reduction of PMMoV was observed, however *E. coli* levels were consistently and significantly (*p* < 0.05) reduced in the filtered (9.59 lnMPN/mL), compared to unfiltered (13.13 lnMPN/mL) water. The die-off intervals of the remaining bacteria were significantly shorter in the filtered (−1.50 lnMPN/day), as compared to the unfiltered (−0.48 lnMPN/day) water. *E. coli* transfer to crop leaves and soils was significantly reduced (*p* < 0.05), as expected. The reduction of *E. coli* in irrigation water and its transfer to crops, by ZVI filtration is indicative of its potential to reduce pathogens in produce pre-harvest environments.

## 1. Introduction

The dependence on immense quantities of water for agricultural irrigation will not decrease in the foreseeable future across the United States. Groundwater, as a limited natural resource, cannot remain as the primary source indefinitely; non-traditional sources of irrigation water, including surface water, will need to be safely implemented as alternatives to groundwater. Maintaining the safety of the food supply through remediation techniques is as significant as preserving the quality of soil and plant health. Our study has significantly contributed to the ZVI-related science with the inclusion of physicochemical analyses, the use of both viral and bacterial organisms, and having been performed in a longitudinal field environment.

Groundwater is generally viewed as a low-risk source of water, however there are situations through which groundwater sources can become microbially contaminated. Murphy et al. [1] reviewed enteric diseases attributed to groundwater. It was determined that over six hundred waterborne outbreaks that occurred over a sixty-five-year period were caused by viral (norovirus and hepatitis A virus), bacterial (*Shigella* and *Campylobacter*), and protozoan (*Giardia*) pathogens. Outbreaks were caused by a variety of bacterial (46%), viral (40%), and protozoan (14%) agents when the pathogen could be confirmed or was suspected (*n* = 169 outbreaks).

Non-traditional sources of irrigation water, such as pond or river surface waters, are considered more susceptible to microbiological contamination than groundwater. Surface water is open to contamination through various point and non-point sources and does not have the beneficial built-in filtration of groundwater. In their review, Ritter et al. determined primary sources of surface water contamination to be by sewage discharge or treatment failure, storm run-off, animal production and crop irrigation [2]. Non-traditional water sources in the Mid-Atlantic region of the United States were surveyed for *Escherichia coli*, total coliforms, and enterococci. These organisms were each detected in more than 85% of the 333 samples tested, and *E. coli* levels were significantly higher during the growing season of May through October, compared to other months [3]. Truitt et al. evaluated Virginian surface waters used for agricultural irrigation during 2015 and 2016; *Salmonella* was detected in 19% of the four hundred samples collected [4]. Campylobacter was detected in Belgian irrigation water and transferred to 9% (*n* = 88) of the raw agricultural commodities, lettuce plants, irrigated with that water [5]. Surface water used for irrigation in Egypt was found to contain enteric viruses (NoV GI, HAV, HAdVs, and RVA) in 84% of the thirty-two samples collected. Subsequently, raw agricultural commodities irrigated with that water were contaminated in 77% of the 128 samples collected by at least one virus [6].

In many studies where enteric pathogens and fecal contamination of water sources are investigated, the primarily focus on one type of organism [3,4,5,6,7]. However, bacteria and viruses are both found in human feces, but their structural differences create variations in the persistence and efficacy of water treatment technologies. De Giglio et al. surveyed Italian groundwater sources for both viral and bacterial contaminants to determine possible connections between fecal indicator bacteria (FIB) and viral pathogens, including norovirus, hepatitis A virus, rotavirus, and enterovirus. Viral pathogen presence was not correlated with FIB, and therefore FIB are not indicative of viral contamination in irrigation water [8]. Pepper mild mottle virus (PMMoV) is widely used as a viral fecal contamination indicator. It is considered to be more representative of viral pathogen presence than bacterial indicators due to the global ubiquity of the virus in human waste, the high concentrations shed, and its biological similarity to enteric viruses [9,10,11,12]. Biological treatments were ineffective in reducing PMMoV concentrations, and additional processing by chlorination yielded a final concentration of more than 4 log copies per liter in the effluent [10]. Successful removal of bacteria through these types of treatments renders FIB testing ineffective [11], while PMMoV remains persistent, detectable, and indicative of potential viral pathogen presence [13].

Zero-valent iron (ZVI) is a type of metallic nanoparticle that can be used as an additive to traditional matrices, such as sand, for the removal of bacterial and chemical contaminants. ZVI can be implemented for the treatment of irrigation water as a low-cost additive, particularly if sand filtration units are already in use. ZVI can be produced through a variety of physical and chemical methods, such as grinding and organic solvents [14]. Zero-valent iron particles are chemically unstable and generate multiple reactive oxygen species (ROS) such as ferrous and ferric ions [15]. ROS produced by ZVI can inactive *E. coli* by causing internal oxidative stress through superoxide formation and suppression of critical gene expression during the exponential phase of growth [16]. Diao et al., 2009 also demonstrated ZVI inactivation of bacteria, *Bacillus subtilis*, through a combination of ROS generation, binding of particles to bacterial surfaces, and membrane disruption, determined through scanning electron microscopy methods [17].

Several studies have been performed evaluating the efficacy of zero-valent iron–sand filtration for the reduction of microbiological organisms in irrigation waters, specifically. *E. coli* O157:H12 populations were reduced significantly more by ZVI–sand filtration than either no filtration or just sand filtration in a study by Ingram et al. in 2012 [18]. ZVI–sand at a 35:65 ratio has been shown to significantly reduce *L. monocytogenes* and *E. coli* levels in irrigation water compared to unfiltered water [19]. Significant enteric virus (AiV, Ad41) removal has been demonstrated by filtration through ZVI–sand compared to filtration through sand alone [20]. Agreeably, Chopyk et al. found an average virus-like particle concentration of 8–9 log particles per milliliter before filtration through ZVI–sand, and a significantly lower post-filtration concentration of 6–7 log particles per milliliter [21].

Although *E. coli* removal by ZVI–sand filtration has been performed previously, this study aimed to address remaining gaps in the knowledge base. The sequential-filter unit was designed to improve the efficacy of bacterial and viral removal from irrigation water while prohibiting iron from leaching into the pre-harvest environment, including water, crops, and soil. Additionally, this study was performed outside of typical laboratory conditions on cucurbit and cruciferous crops, which are susceptible to contamination due to their proximity to the ground. The persistence of *E. coli* was also monitored in irrigation water exposed to environmental conditions after passage through the filtration unit. These parameters within the study provide a data set which is more likely to resemble the conditions within a pre-harvest environment.

## 2. Materials and Methods

### 2.1. Experimental Design

**Figure 1:**
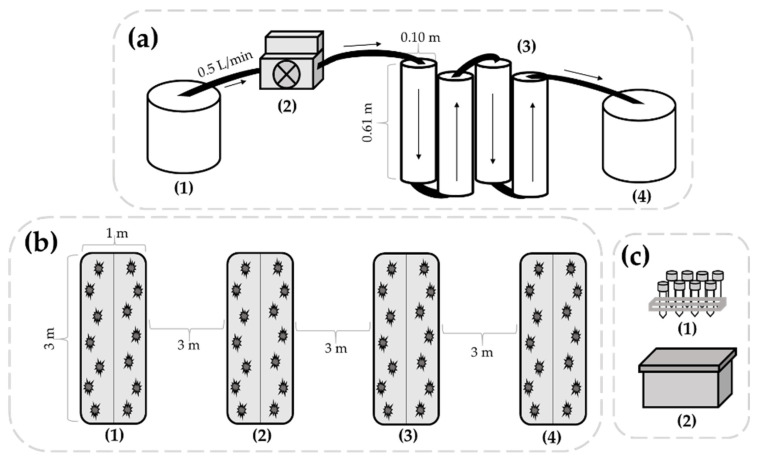
Schematics of the water filtration unit (**a**), agricultural plots (**b**), and water sample storage area (**c**) used in this study. Filtration (**a**) was performed by collecting irrigation water in a sterile container (1), connecting the peristaltic metering pump (2), and pumping the water through the filtration unit (3) into the collection container (4). The direction of water movement is indicated by the arrows. The agricultural plots (**b**) were each 3 × 1 m in size, with 3 m buffer areas between plots. Plots (1) and (2) were amended with composted and raw poultry litter, respectively, and irrigated with filtered water throughout the trial. Plots (3) and (4) were amended with composted and raw poultry litter, respectively, and irrigated with unfiltered water throughout the trial. Irrigation water (**c**) for the evaluation of bacterial persistence was stored in sterile centrifugal tubes (1), inside a secondary container (2), located adjacent to the agricultural plots.

ZVI-filtered and unfiltered water were used for agricultural irrigation over a 60-day period from August through October. Water was inoculated with *E. coli* TVS 353, a non-pathogenic strain, prior to each filtration event throughout the trial, and spray-irrigated onto four 3 m^2^ plots (Figure 1b). Cucurbit (cucumber) plants were transplanted to the plots prior to this study and leaves were analyzed for *E. coli* levels for the first 10 days prior to harvesting the fruits. A mix of cruciferous crops (broccoli, Brussels sprouts, cabbage, cauliflower, and kale) were transplanted to plots after the initial harvest, and leaves were analyzed for *E. coli* levels for the last 20 days of the study. The removal and persistence of the bacterium was monitored in water as well as the transfer to crop leaves. Water and soil physicochemical parameters were monitored in addition to atmospheric parameters including daily temperature (°C) and rainfall (in) values.

### 2.2. Construction of Filtration Apparatus

The filtration unit was constructed using polyvinyl chloride (PVC) pipes and fittings connected by PEX cross-linked polyethylene tubing (SharkBite, Cullman, AL, USA). As shown in Figure 1a, unfiltered water was stored in a sterile container (1) and water was pumped through the filtration unit at a rate of 0.5 L/min using a 25.3 GPH standard peristaltic metering pump (2) (Cole Parmer, A3 Series). Four filtration columns were aligned vertically to allow water to flow through sequentially (3). Each column (4 in diameter × 2 ft length PVC) was filled with either 100% sand (first and fourth columns), or a homogenous blend (50:50) of sand and zero-valent iron (second and third columns). The first column with sand was designed to remove large particles within the water and increase potential contact between the bacterial contaminants in the second and third ZVI and sand columns. The final column of sand was implemented to prevent any potentially dislodged iron particles from leaving the filtration unit. Sand (Northern Filter Media, Muscatine, IA, USA) and ZVI (Peerless Metals, Detroit, MI, USA) particles were prepared as previously described [22], and the filtration unit had a total pore volume of 10 L. Water was collected in an additional sterile container (4) prior to irrigation and sample collection.

### 2.3. Collection and Management of Water

Water was collected from a pond in the Mid-Atlantic region of the United States. This site, MA11, was previously characterized by the CONSERVE team and is currently used as a non-traditional source of agricultural irrigation water [3,21,23]. The water was collected prior to each irrigation event (days 0, 10, 20, 30, 40, and 50) and transported to the laboratory. Water was diluted 10-fold in sterilized Milli-Q Ultrapure water. Additionally, 1 L volumes of water from MA11 were stored at refrigeration temperature to be used to flush the filtration systems every 48 h, using 15 L volumes, between irrigation events.

### 2.4. Preparation and Inoculation of E. coli TVS353

*E. coli* TVS 353, a strain originally isolated from an agricultural water source in Salinas Valley, California, was selected for this study due to its robust nature and ability to persist in the environment [24]. *E. coli* TVS 353 is rifampicin-resistant which allows for simplified isolation and enumeration from the complex environmental matrices analyzed in this study [25,26]. *E. coli* TVS 353 was inoculated into the water samples resulting in a final level of 13.13 ± 0.90 lnMPN/mL. The inoculum level of ~13 lnMPN/mL (5 logMPN/mL) was selected based on the microbial data previously collected for the site MA11. A total of 33 samples was obtained and processed for total coliform (TC) levels between September 2016 and October 2018 with a maximum concentration of 5.12 logMPN/100 mL [3]. The inoculum level was set to exceed the maximum level of *E. coli* observed to determine if the filtration techniques were effective in a worst-case scenario. This inoculum level also allowed for an evaluation of *E. coli* transfer from water to crop leaves and of *E. coli* persistence in soils irrigated with filtered or unfiltered water.

### 2.5. Physicochemical and Atmospheric Data Collection

Filtered and unfiltered water samples (*n* = 168) were collected for triplicated physicochemical analysis on the day of each irrigation event. Iron (mg/L), pH, and hardness (mg/L) levels were monitored using a Hach test kit (Model HA-62B). The Hach test kit was selected for its inclusion of the FerroVer reagent used for evaluation of overall iron content including ferrous and ferric ions, soluble iron compounds, and insoluble iron forms. Dissolved oxygen (%) was evaluated using a YSI-ProDSS probe. The temperature (°C) of the stored water samples and the relative humidity (%) were recorded using the Simbow GSP-6 data logger hourly throughout the duration of the study. Air temperature (°C) and precipitation (in) data were obtained from the Delaware Environmental Observing System station located on the Newark, DE Agricultural Farm. Analyses of soil samples were performed on samples collected before and after irrigation by the University of Delaware Soil Testing Laboratory. Parameters tested included moisture content, pH, carbon:nitrogen ratio, and micronutrient levels (B, Ca, Cu, Fe, K, Mg, Mn, P, S, and Zn).

### 2.6. Application of Irrigation Water

Filtered or unfiltered water was applied to two plots (3 m × 1 m) each by spray irrigation immediately following filtration. Plots were amended with raw poultry litter or composted poultry litter (~6.5 lb/plot) and each received one type of water, filtered or unfiltered. A total of 4 L of water was applied to each 3 m^2^ plot resulting in approximately 285 mL per cucumber plant area (14 plants/plot) and 200 mL per cruciferous plant area (20 plants/plot) during each irrigation event. Plots which initially received filtered or unfiltered water only received that water type during irrigation events and irrigation was supplemented with groundwater via driplines between those events. Each plot was irrigated six times (once every 10 days) over the course of the study. A visual representation of the agricultural plots described above is provided in Figure 1b.

### 2.7. Collection of Samples

#### 2.7.1. Water

The samples, 30 mL of filtered or unfiltered water, were collected (*n* = 168) following each filtration event (*n* = 6). Day 0 samples were stored at refrigeration temperature until microbial analyses were performed. Samples were also collected on days 1, 3, 5, 7, and 10 after each irrigation event from a secondary container located adjacent to the agricultural plots where they were stored, as depicted in Figure 1c. Water samples were removed at each sampling point and transferred to sterile Whirl-Pak bags for bacterial enumeration.

#### 2.7.2. Soil and Crop Leaves

Soil samples (*n* = 24) were collected from six locations in each plot after each irrigation event (*n* = 6). Samples were homogenized, and 30 g subsamples were transferred to sterile Whirl-Pak bags for bacterial enumeration. Crop leaf samples (*n* = 40) were collected from cucurbit (cucumber) or cruciferous (Brussels sprouts, kale, cauliflower, cabbage and broccoli) plants in each plot after irrigation events (*n* = 3) on days 0, 40, and 50. Leaves were also collected 1, 3, and 5 days after each irrigation event, on days 1, 3, 5, 41, 43, 45, 51, 53, and 55. On every sampling day, a total of 12 crop leaves were collected from 6 plants for each treatment type and placed in sterile Whirl-Pak bags for bacterial enumeration.

### 2.8. Isolation and Enumeration of E. coli TVS 353

Water samples were processed in 30 mL volumes while leaf samples were weighed, and the total sample was processed. A 1:5 dilution of each sample was performed using tryptic soy broth supplemented with 80 µg/mL rifampicin (TSBR); 120 mL of TSBR was added to water samples and the volume was added to leaf samples varied to obtain a 1:5 dilution (*w*/*w*). Samples were vigorously hand massaged for two minutes prior to the transfer of the suspension to 48-well deep well plates (Millipore Sigma-Aldrich; Darmstadt, Germany) containing 1× TSBR. Serial dilutions (4 wells/dilution) using a multi-channel pipet were performed in quadruplicate for each sample and then incubated at 37 °C for 24 h. A 5 µL aliquot from each well was channel-streaked on tryptic soy agar supplemented with 80 µg/mL rifampicin (TSAR) for the presence of *E. coli* TVS 353. Plates were incubated at 37 °C for 24 h. Results were recorded as positive or negative for growth.

### 2.9. Reduction of PMMoV in Irrigation Water

Samples of filtered and unfiltered water were collected from three of the irrigation events in 50 mL volumes, each in triplicate (*n* = 18). The samples were concentrated using Centricon 100 kDa filters (Millipore Sigma-Aldrich) via centrifugal ultrafiltration. Viral RNA was extracted (AllPrep PowerViral DNA/RNA Kit; Qiagen; Hilden, Germany) and detection was performed using a real-time quantitative PCR (RT-qPCR) molecular assay. The primer-probe set [27,28] was selected for use with the Rotor-Gene Q apparatus (Qiagen), based on the available research of successful PMMoV detection in environmental waters.

### 2.10. Data Analysis

Data were analyzed, and figures and tables were generated, using Microsoft Excel and JMP 15 Pro statistical software. Physicochemical parameter data from water and soil samples were analyzed using analysis of variance (ANOVA) and *t*-test analyses for comparison between treatment groups; Standard least squares regression and multiple analysis of co-variance (MANCOVA) were used to analyze time-series data.

Positive and negative results from the *E. coli* enumerations were uploaded to a Microsoft Excel-based calculator [29] to determine populations of *E. coli* TVS 353 as most probable numbers (MPN). Populations of TVS 353 in irrigation water and on leaves were calculated using MPN values generated in Excel. *E. coli* TVS 353 data from water and crop leaves were compared by treatment type using ANOVA and t-test analyses. Decay rates of *E. coli* TVS 353 in water were calculated using the equation:

*r* = [ln(MPN·mL^−1^*_t_*) − ln(MPN·mL^−1^_0_)]/(*t_t_* − *t*_0_) (1)

which was modified from the equation presented by Anderson et al., 2005 [30]. In this equation, *r* = bacterial rate of decay, 0 = the initial enumeration on the day of the irrigation event, and *t* = time between the initial and final bacterial enumerations (days). The final bacterial enumeration was considered as either the day bacterial levels reached the limit of detection (1.6 lnMPN/mL) or at the final sampling prior to the next irrigation event if levels remained above the limit of detection. The transfer and persistence of *E. coli* TVS 353 were calculated individually after each irrigation event and then combined for presentation in the tables and figures. The statistical threshold for all analyses was set to *p* = 0.05, and all *p*-values less than this threshold were considered to be significant.

PMMoV detection data were recorded as genomic copies per ml and cycle threshold (CT) values. RT-qPCR was performed over 40 cycles, and genomic amplification above the threshold prior to the fortieth cycle was considered positive detection. For results consisting of positive and negative detection, delta CT values were used for analyses. dCT values were calculated using the difference between the CT value and the terminal cycle (40), where larger dCT values indicated a larger concentration of genomic copies in a reaction.

## 3. Results

### 3.1. Effects of ZVI–Sand Filtration on Water Quality

The pH, dissolved oxygen (%), hardness (mg/L), and iron (mg/L) levels of irrigation water with and without ZVI–sand filtration were recorded in triplicate after each filtration event and compiled for analyses. pH was significantly (*p* < 0.05) lower in unfiltered water (6.89 ± 0.68) than filtered water (8.82 ± 0.71). Dissolved oxygen was significantly higher in unfiltered water (9.17% ± 0.69%) than filtered water (4.56% ± 2.25%). Hardness and iron levels were below the detection levels of 17.1 mg/L and 0.5 mg/L, respectively for all samples tested (*n* = 36).

### 3.2. Reduction of E. coli in Irrigation Water and Transfer to Crop Leaves

*E. coli* TVS 353 populations were significantly reduced (*p* < 0.05) by ZVI–sand filtration with reductions of 2.78–4.78 lnMPN/mL observed over the six irrigation events. As expected, due to the lower concentration of *E. coli* TVS 353 observed in filtered water, transfer of *E. coli* to crop leaves and soil was significantly reduced (*p* < 0.05) after irrigation with filtered water compared to unfiltered water. The reduction in transfer of *E. coli* to crop leaves after irrigation filtered water, compared to unfiltered, ranged from 2.99–7.46 lnMPN/g, while the reduction in transfer to soil ranged from 2.19–6.43 lnMPN/g. The average concentrations of *E. coli* TVS 353 (MPN/mL) in the unfiltered and filtered irrigation water, along with the average reductions of *E. coli* in water and the subsequent transfer to crop leaves and soil for each irrigation event monitored are shown in Table 1.

### 3.3. Prolonged Effects of ZVI–Sand Filtration on Bacterial Survival

Bacterial persistence was monitored in filtered and unfiltered irrigation water to evaluate whether contact with the zero-valent iron within the filtration unit resulted in any measurable prolonged effects. Water samples were collected on the day of filtration and 1-, 3-, 5-, 7-, and 10-days post-filtration. The initial concentrations of *E. coli* TVS 353 in filtered (9.59 ± 1.13 lnMPN/mL) and unfiltered (13.13 ± 0.90 lnMPN/mL) water were significantly different (*p* < 0.05) on day 0 (enumeration performed immediately after filtration). The bacterial rates of decay (r) were determined both graphically and algebraically.

**Figure 2:**
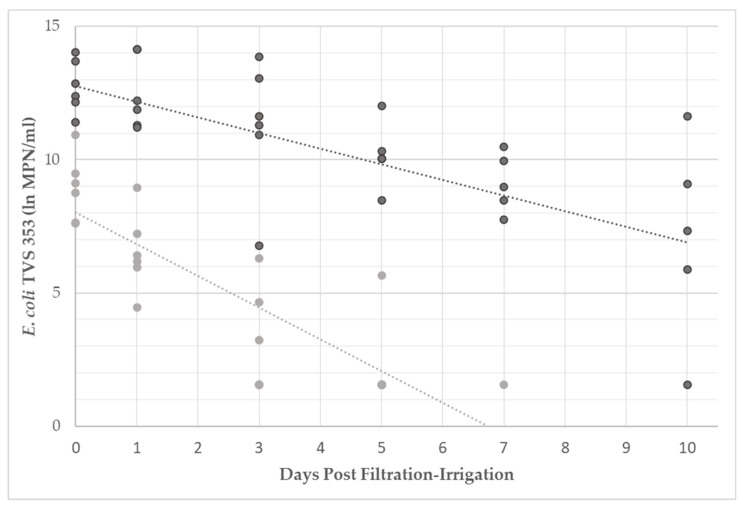
Levels of *E. coli* TVS 353 (lnMPN/mL) in filtered (light gray) and unfiltered (dark gray) irrigation water. Samples were collected the day of filtration-irrigation and 1, 3, 5, 7, and 10 days afterwards. When bacterial enumeration resulted in levels below the detection limit, values of 1.54 lnMPN/mL were used for analyses and display within the figure.

A hraphical representation of bacterial decay is presented in Figure 2 for *E. coli* TVS 353 in filtered and unfiltered water. Bacterial levels for all filtration events (*n* = 6) are presented. If bacterial levels were below the detection limit of 1.61 lnMPN/mL, 1.54 lnMPN/mL (the greatest value possible below the detection limit) was used for analyses and visualization in the figure. Bacterial concentrations were reduced to the detection limit in unfiltered water by day 5 during the third irrigation event, and by day 10 during the fourth irrigation event. Bacterial concentrations were at the detection limit in filtered water by day 3 in the first three irrigation events, by day 5 in the fourth and sixth, and by day 7 for the fifth; no positive detection occurred on days 7 or 10 for any *E. coli* TVS 353 in filtered water. The trendlines were used for graphical calculation of the average rate of decay in filtered and unfiltered water. *E. coli* TVS 353 decayed at a rate of −0.59 lnMPN/day (R^2^ = 0.42) in unfiltered water and −1.19 lnMPN/day (R^2^ = 0.64) in filtered water.

The data in Figure 2 were compared by the treatment group using MANCOVA analysis. *E. coli* TVS 353 levels were determined to be significantly different (*p* < 0.05) between treatments on day 0, which agreed with the results from the previous analyses performed. *E. coli* levels significantly decreased (*p* < 0.05) over the 10-day period in both the filtered and unfiltered water. The rate of decay in the filtered water was significantly (*p* < 0.05) greater than in the unfiltered water.

Algebraic analyses of bacterial decay rates were calculated using Equation (1), and displayed in Table 2. The decay of TVS 353 was greater in filtered water for all six filtration-irrigation events, ranging from −0.63 to −3.13 lnMPN/day, and averaging −1.49 ± 1.07 lnMPN/day. Decay in unfiltered water ranged from −0.05 to −0.94 lnMPN/day, averaging −0.48 ± 0.31 lnMPN/day. However, a statistical comparison between the two treatment groups by *t*-test resulted in no significant difference (*p* = 0.068).

### 3.4. Effects of ZVI–Sand Filtered Irrigation Water Application on Soil Quality

Soil in plots irrigated with filtered and unfiltered water was monitored throughout the 60-day study. The physicochemical properties of the soil were analyzed before and after each of the irrigation events to determine if any immediate or prolonged effects could be observed. The parameters monitored were selected for their potential to influence crop growth and overall soil health and included moisture content, pH, carbon-nitrogen ratio, and the presence a number of significant minerals. The minimum, maximum, and mean values recorded are displayed in Table 3, along with the *p*-values from the MANCOVA analyses performed by parameter across treatment groups. There were no changes in physicochemical parameters observed over the course of the study that were significantly different (*p* > 0.05) between soils irrigated with filtered or unfiltered water.

### 3.5. Reduction of PMMoV in Irrigation Water

**Figure 3:**
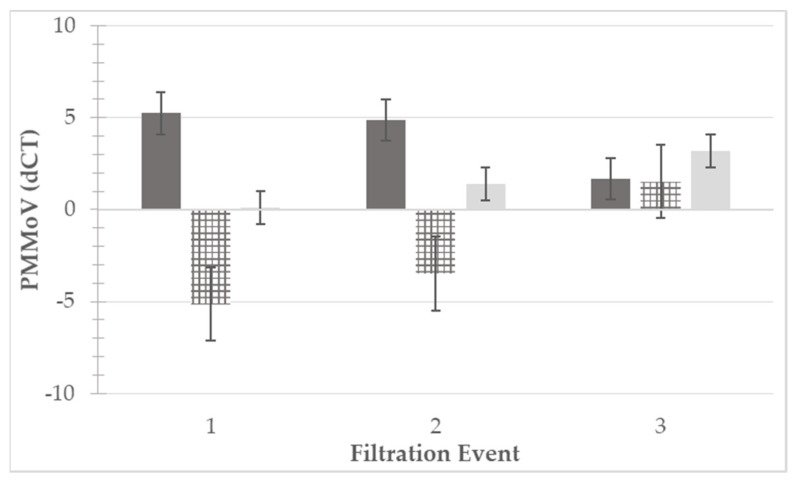
Pepper mild mottle virus (PMMoV) levels detected via RT-qPCR molecular assay. PMMoV levels were quantified for three filtration events in unfiltered (dark gray, left) and filtered (light gray, right) irrigation water. Differences in levels between unfiltered and filtered water from each filtration event are displayed as the dark and light gray hatched bars (center). Quantification was performed using delta cycle threshold (dCT) values, with 40 cycles being the terminal value and the dCT being the difference between the terminal value and the detected value (e.g., CT 32, dCT = 40 − 32 = 8). Higher dCT values represented higher amounts of viral RNA present within the reaction. Standard error bars are presented for each bar.

PMMoV was detected in 12/18 (67%) and 7/18 (39%) of the RT-qPCR reactions for unfiltered and filtered water samples, respectively. Of the reactions with successful detection, unfiltered water had an average of 32 copies/reaction, and filtered water had an average of 13 copies/reaction. Due to the low positivity rate in the filtered samples, dCT was also used for comparative analysis, data are presented in Figure 3. Of the three filtration-irrigation events in which PMMoV detection was performed, two had significantly higher (*p* < 0.05) dCT values in the unfiltered compared to filtered water, while there was no significant difference (*p* = 0.353) observed in one. Overall analysis of dCT values (*n* = 18) between the two treatment groups was performed using one-way ANOVA, and the dCT values of the unfiltered water samples were significantly higher (*p* < 0.05) than those of the filtered water samples. PMMoV was most efficiently reduced in Event 1, with a reduction of 5.14 dCT, followed by Events 2 (3.48 dCT) and 3 (1.52 dCT).

## 4. Discussion

Levels of *E. coli* TVS 353 in irrigation water were successfully reduced by zero-valent iron–sand filtration compared to unfiltered water. Previous studies have illustrated the effectiveness of zero-valent iron treatments, including a 2019 study where both sand and ZVI–sand filtration were performed and compared to untreated irrigation water [15]. Sand and ZVI–sand filtration significantly reduced *L. monocytogenes* and *E. coli* populations compared to unfiltered water. Additionally, the Gram-positive *L. monocytogenes* levels were significantly lower after ZVI–sand filtration, as compared to sand filtration alone. Comparatively, this study investigated the more robust Gram-negative *E. coli* and also demonstrated a significant reduction in *E. coli* levels after ZVI–sand filtration.

The study by Kim et al., 2020 also evaluated the efficacy of ZVI–sand filtration for the removal of *E. coli* [22]. Significantly greater reductions in *E. coli* populations were observed using ZVI–sand filtration, as compared to sand filtration. Consequently, the *E. coli* levels transferred to crops and soil were significantly lower after ZVI-sand-filtered irrigation than with sand-filtered alone or with unfiltered irrigation water. Agreeably, in our study, the transfer of *E. coli* to cruciferous and cucurbit crop leaves was lower after irrigation with filtered water compared to unfiltered water, when applied using the same spray-irrigation techniques. This finding was expected, given that the initial bacterial concentrations were significantly lower in the filtered water. However, these findings were denoted as an important confirmatory part of the investigation, they agree with the findings of Ingram et al., and they complement the work performed by Marik et al. [17,18].

The significant decrease in dissolved oxygen and increase in pH of water after filtration was likely due to the multitude of reactions occurring between the water and iron particles. Cheng et al., documented the formation of ammonia from nitrates in water when zero-valent iron mesh was introduced, which resulted in a rise of pH levels [31]. Ryu also observed nitrate reduction to ammonium using zero-valent iron [32]. Higher dissolved oxygen concentrations have been shown to increase reactions between nitrates in water and zero-valent iron particles, and dissolved oxygen levels were reduced after the introduction of ZVI particles [33], as demonstrated also in this study.

The absolute elimination of all bacterial contaminants is unlikely in a water source, particularly in variable environmental waters. Sand filtration of drinking water can decrease bacterial population by 1.3 log/mL [34], however sand alone does not provide prolonged effects for control of the remaining bacterial populations. The persistence of the remaining *E. coli* TVS 353 populations in filtered and unfiltered irrigation water was evaluated, and decreased persistence was observed in filtered waters. The authors acknowledge the variability observed in persistence across irrigation events, as this study was performed in a natural growing environmental and lacked the controlled climate of a laboratory setting. Thus, temperature, UV index, humidity, precipitation and other factors likely impacted bacterial persistence and varied within each of the 10-day periods. Ingram et al., evaluated the reduction of *E. coli* in irrigation water using ZVI–sand and sand filtration along with the persistence of the bacteria in water following the filtration events. The persistence of *E. coli* after passage through ZVI–sand filters was significantly lower than that of bacteria in water filtered through sand or left unfiltered [18]. Marik et al. performed a similar study, as discussed above, and included an investigation of bacterial persistence on crop leaves. No significant differences were observed in the persistence of *E. coli* on crops leaves irrigated with ZVI–sand and sand filtered water. However, *L. monocytogenes* did experience reduced persistence on leaves after ZVI–sand filtration, as compared to sand filtration [19].

Han, Huang, and Liu utilized microscopy techniques to evaluate the mechanisms by which bacteria were adsorbed to zero-valent iron particles or inactivated by contact with the particles [35]. Adsorption was found to be the primary mechanism by which bacteria were removed from media. However, the bacteria which made contact with the iron particles experienced damage to the cell membranes. Additionally, increasing the contact time between the bacteria and the iron particles increased this damage, and led to inactivation of the bacteria. This study used a consistent flow of water through the filtration unit, and therefore did not examine bacterial contact times of 120 min, as performed in the work by Han, Huang, and Liu. The lengthy period used was not conducive to the filtration of irrigation waters. However, the decrease in persistence observed after passage through the ZVI–sand filtration unit in our study demonstrates that minimal contact time can provide enough damage to the bacterial membranes for significant impacts to be observed.

The sand filtration used techniques for drinking water treatment have been shown to significantly remove bacterial contaminants while PMMoV remained detectable in the effluent [36]. Viruses are smaller in size compared to bacteria and can escape through pores without making contact or becoming lodged within the filtration unit. However, viruses can greatly vary in shape and size, which has been shown to impact the efficacy of sand filtration [37]. Larger viruses, such as the bacteriophage T4, approximately 200 nm in size, can be removed by sand filtration more effectively than MS2, which is approximately 30 nm in size. This may support the variability observed in this study and the comparison to other studies utilizing ZVI–sand filtration for viral removal. Shearer and Kniel studied the impacts of ZVI–sand filtration on the removal of Tulane virus and murine norovirus, two commonly used surrogates for enteric viruses. Both viruses are icosahedral in shape, and approximately 36 nm in diameter. A 2–3 log greater reduction of viral particles was observed in water filtered through ZVI–sand compared to sand [38].

In this study, we did not observe large reductions of PMMoV, and the efficacy of filtration varied amongst the three filtration events. This may be due to the lower levels of PMMoV observed in the initial, unfiltered water and the sporadic detection frequency observed. However, PMMoV is a rod-shaped plant virus with dimensions of approximately 18 nm by 300 nm, and has been shown to be less affected by filtration methods than icosahedral-shaped enteric viruses [39].The surface charge of PMMoV may have also differed from enteric viruses in irrigation waters with neutral pH. The isoelectric point of PMMoV is approximately 3.7–3.8 [40], while enteric viruses tend to have isoelectric points closer to neutral pH, such as norovirus with its PI of 5.9–6.0 [41]. The potential difference in surface charges could also alter the reactions between viruses and the chemical compounds within the water or the iron particles themselves.

## 5. Conclusions

Filtration through a zero-valent iron-sand filtration unit can significantly reduce the presence of pathogenic bacterial contaminants in irrigation water, as demonstrated here using *E. coli* TVS 353. The subsequent reduction in the transfer of bacterial contaminants to raw agricultural commodities suggests this risk to public food safety could potentially be reduced through the implementation of a ZVI–sand filtration of irrigation water. Additional research is needed to evaluate the capacity and longevity of ZVI–sand filtration units, particularly for use with inherently variable surface and reclaimed waters. The removal of viral contaminants, including human, plant, and animal pathogens, has not been consistently demonstrated in the study, and the structure, concentration, and interactions with ZVI should be evaluated to determine the cause. Correlations between dissolved oxygen, pH, PMMoV and *E. coli* removal efficacy were not investigated due to the longitudinal nature of this study. Further evaluation of all parameters discussed here, in large, simultaneously monitored, sample populations to provide insight into the relationships and factors impacting filter efficacy is warranted.

## Figures and Tables

**Table 1:** Average concentrations (lnMPN/mL) of *E. coli* TVS 353 in unfiltered and filtered irrigation water, and average transfer to leaf and soil (lnMPN/g) for each irrigation event. Reductions and differences between unfiltered and filtered water for each irrigation event in irrigation water, on leaves, and in soil, with the overall average for all irrigation events, in all matrices.

Irrigation Event	Water			Leaf			Soil		
	Concentration ^b^		Reduction	Transfer ^c^		Reduction	Transfer ^c^		
	Unfiltered	Filtered		Unfiltered	Filtered		Unfiltered	Filtered	Reduction
1	11.41	7.65	3.76	10.99	7.38	3.61	7.83	5.63	2.19
2	13.70	10.92	2.78	--- ^a^	---	---	10.56	5.91	4.66
3	12.39	7.61	4.78	---	---	---	9.82	5.95	3.87
4	12.16	8.74	3.42	---	---	---	10.75	5.33	5.42
5	14.02	9.47	4.55	11.70	4.24	7.46	13.32	7.16	6.16
6	12.86	9.12	3.74	8.94	5.95	2.99	11.79	5.35	6.43
Average	13.13	9.89	3.54	11.04	6.53	4.51	11.85	6.12	5.73

^a^ Dashed line indicates no data were available. ^b^ Bacterial concentrations in water are presented as lnMPN/mL. ^c^ Bacterial transfer to leaf and soil is presented as lnMPN/g.

**Table 2:** The average decay (lnMPN/day) of *E. coli* TVS 353 in unfiltered and filtered irrigation water for each irrigation event (*n* = 6) and the difference observed in the bacterial decay between each water type.

Irrigation Event	Decay Rate (r) by Water Type ^1^		Difference ^2^
	Unfiltered	Filtered	Between Rates
1	−0.56	−2.04	1.48
2	−0.64	−3.13	0.49
3	−0.94	−2.02	1.08
4	−0.46	−0.63	0.17
5	−0.22	−0.49	0.28
6	−0.05	−0.66	0.60
Average	−0.48	−1.49	1.02

^1^ Decay rates represent bacterial reduction (lnMPN/mL) per day; e.g., −2.04 represents 2.04 lnMPN/mL decrease per day. ^2^ Difference between decay rates is presented as the absolute value of the differences between the two rates: filtered and unfiltered.

**Table 3:** Physicochemical parameters of soil in plots irrigated with unfiltered and filtered water collected before and after each irrigation event. The minimum, mean, and maximum values of parameters are displayed by irrigation water type.

Soil Parameter (Units)	Water Type	Minimum	Mean	Maximum	*p*-Value
Moisture Content (%)	Unfiltered	1.4	11.6	20.4	0.887
	Filtered	1.4	12.0	18.3	
pH	Unfiltered	5.6	6.2	6.9	0.138
	Filtered	5.8	6.3	6.7	
Boron (ppm)	Unfiltered	0.30	0.48	0.75	0.750
	Filtered	0.32	0.49	0.95	
Calcium (ppm)	Unfiltered	719.26	1086.51	1570.29	0.821
	Filtered	732.45	1056.06	1414.68	
Copper (ppm)	Unfiltered	1.69	2.35	3.39	0.338
	Filtered	3.09	3.84	5.01	
Iron (ppm)	Unfiltered	114.23	134.76	152.97	0.513
	Filtered	104.54	122.12	150.59	
Magnesium (ppm)	Unfiltered	146.65	199.55	291.77	0.779
	Filtered	157.20	216.08	333.20	
Manganese (ppm)	Unfiltered	35.49	41.21	47.61	0.495
	Filtered	40.76	44.48	50.55	
Phosphorous (ppm)	Unfiltered	53.50	110.59	176.76	0.952
	Filtered	90.25	153.76	341.11	
Potassium (ppm)	Unfiltered	221.80	319.74	430.97	0.500
	Filtered	212.81	330.47	626.40	
Sulfur (ppm)	Unfiltered	17.02	47.02	134.27	0.841
	Filtered	21.70	54.98	163.39	
Zinc (ppm)	Unfiltered	2.90	5.71	10.10	0.493
	Filtered	4.20	7.41	11.74	
Carbon:Nitrogen Ratio	Unfiltered	8.80	10.59	11.80	0.936
	Filtered	8.40	10.57	13.20	
